# Introducing the national COPD resources and outcomes project

**DOI:** 10.1186/1472-6963-9-173

**Published:** 2009-09-24

**Authors:** Robert A Stone, Brian DW Harrison, Derek Lowe, Rhona J Buckingham, Nancy A Pursey, Harold SR Hosker, Jonathan M Potter, C Michael Roberts

**Affiliations:** 1 Clinical Effectiveness and Evaluation Unit, Royal College of Physicians, London, UK; 2 The British Thoracic Society, London, UK

## Abstract

**Background:**

We report baseline data on the organisation of COPD care in UK NHS hospitals participating in the National COPD Resources and Outcomes Project (NCROP).

**Methods:**

We undertook an initial survey of participating hospitals in 2007, looking at organisation and performance indicators in relation to general aspects of care, provision of non-invasive ventilation (NIV), pulmonary rehabilitation, early discharge schemes, and oxygen. We compare, where possible, against the national 2003 audit.

**Results:**

100 hospitals participated. These were typically larger sized Units. Many aspects of COPD care had improved since 2003. Areas for further improvement include organisation of acute care, staff training, end-of-life care, organisation of oxygen services and continuation of pulmonary rehabilitation.

**Conclusion:**

Key Points: positive change occurs over time and repeated audit seems to deliver some improvement in services. It is necessary to assess interventions such as the Peer Review used in the NCROP to achieve more comprehensive and rapid change.

## Background

The 2003 RCP/BTS Chronic Obstructive Pulmonary Disease (COPD) Audit demonstrated significant variations in both quality and organization of COPD care, although there were improvements on previous results [1]. Specific areas of care still required attention (most notably the availability of non-invasive ventilation and provision of pulmonary rehabilitation) and so, in tandem with publication of the results, the audit team undertook a series of feedback meetings to Regional Thoracic Society meetings, in the hope this might lead to change.

However, having considered the data from two cycles of National COPD audit it has become clear that repeated audit and time alone may fall short of delivering a desirable consistency and penetration of change. We therefore designed the National COPD Resources and Outcomes Project (NCROP) to go a step beyond audit alone in assessing how a Peer Review Intervention might affect change in COPD care amongst participating hospitals through the lifetime of an audit cycle. The NCROP is a collaborative study between three partner organizations, the Royal College of Physicians of London (RCP), The British Thoracic Society (BTS) and the British Lung Foundation (BLF) and is running over a 4 year period. It has three phases, the first of which, an initial survey of participating Units, we report here. Phases 2 and 3, the Peer Review intervention and repeat of the baseline assessment, will be reported later.

## Methods

The baseline survey (2007) in Phase 1 of NCROP was designed to assess the general organization of COPD care and that relating to specific indicators in the 100 participating sites at the beginning of the 4 year period. The study was approved by the Medical Research Ethics Committee of the University of London. The survey questionnaire was split into five domains. Organisational questions were derived largely from those asked in the 2003 RCP/BTS COPD audit [1]. Further questions were compiled around the Key Performance Indicators (KPI), developed from existing national BTS/NICE guidelines and following consultation with an expert multi-disciplinary panel. For each quality indicator the NCROP units were asked to indicate whether they 'fully met' the indicator, 'partially met' it or whether it was 'not met at all'. The survey questions overall were grouped into 5 main areas;-

### 1. Organisational aspects of COPD service

Questions focussed on size of unit, numbers of admissions, acute admissions practices, bed management, triage, medical and specialist nursing staff numbers, management of critically ill cases and adherence to BTS/NICE COPD management guidelines.

### 2. NIV

Questions focussed on availability, service governance, staff training, equipment, patient information and adherence to BTS guidelines.

### 3. Pulmonary rehabilitation

Questions focussed on availability, frequency, adherence to recommended guidelines, governance, staffing and funding.

### 4. Early discharge schemes

Questions focussed on availability, service governance, patient information, data collection, communication with primary care and adherence to BTS management guidelines,

### 5. Oxygen service

Questions focussed on availability of both long-term and ambulatory oxygen provision, patient education, patient information and adherence to BTS guidelines.

To assess representation of NCROP participation in 2007 the 100 NCROP units were compared against 201 that were eligible but did not participate. The comparison data used was that from participants in the last National COPD audit in 2003, the methods of which have been published elsewhere [1].

There were 87 NCROP sites that had also participated in the 2003 national COPD audit, thereby allowing a comparison of change in the organization of care between 2003 and 2007 in those items measured in both. Change in the percentage of sites having desirable features of organization was tested for statistical significance using McNemars test.

## Results

Of a possible 301 Acute UK NHS Healthcare Trusts, 100 hospitals agreed to participate in the NCROP. Of these, 81 were in England, 8 in Scotland, 6 in Wales and 5 in Northern Ireland. Of 201 eligible but not participating, 158 (79%) were in England, 19 (9%) in Scotland, 14 (7%) in Wales and 10 (5%) in Northern Ireland.

87 (87%) of the NCROP sites had also taken part in the 2003 COPD audit, compared to 135 (67%) of NCROP non-participants. NCROP participants were generally bigger hospitals in regard to total number of hospital beds in 2003 and COPD patients admitted in 2002 (Table 1). There was little difference in respiratory consultants per 1000 admissions or per 1000 beds nor in regard to Trust catchments (results not given). The available data from 2003 participants suggests that NCROP participants were generally better organised for taking care of their COPD patients than NCROP non-participants (Table 1).

**Table 1:** 2003 national audit results for NCROP participants and non-participants

**INDICATOR (2003 audit)**	**NCROP** **PARTICIPANTS**		**NCROP** **NON-PARTICIPANTS**	
Participation in 2003 audit	87%	(87/100)	67%	(135/201)

Beds (2003)	Median 593 IQR 438-807 N = 87		Median 531 IQR 398-736 N = 135	

COPD Patients (2002)	Median 545 IQR 345-754 N = 86		Median 414 IQR 302-694 N = 128	

Respiratory consultants per 1000 admissions	Median 4.0 IQR 2.3-5.8 N = 86		Median 4.0 IQR 2.0-6.6 N = 129	

Respiratory consultants per 1000 beds	Median 3.4 IQR 2.5-4.4 N = 87		Median 3.6 IQR 2.4-4.8 N = 134	

Two post take rounds	70%	(61/87)	53%	(68/128)
Ward-based system	62%	(53/86)	57%	(76/134)
Specialty triage	41%	(36/87)	28%	(37/134)
Respiratory Ward	76%	(66/87)	58%	(78/134)
Access to respiratory nurse	71%	(62/87)	71%	(95/134)
NIV availability	97%	(84/87)	94%	(126/134)
NIV on ICU	68%	(59/87)	54%	(72/134)
NIV on wards	70%	(61/87)	58%	(78/134)
Formal pulmonary rehab programme	71%	(62/87)	58%	(78/134)
Access to early discharge scheme	46%	(40/87)	44%	(59/134)
Written local guidelines for assessment of COPD	60%	(52/87)	55%	(72/131)

There were clear improvements in COPD service provision from 2003 to 2007 amongst the 87 NCROP hospitals that had also participated in the earlier audit (Table 2). Most notably, early discharge services had increased from 46% to 63% of units. The use of specialty triage remains poor at 59%, despite specialty ward provision improving from 77% to 87%.

**Table 2:** 2003 national audit and 2007 NCROP survey results from Hospitals participating in both surveys

**INDICATOR**	2003 DATA*		2007 DATA*		**McNemars Test P value**
Written local guidelines	60%	52/87	76%	66/87	0.024
Specialist Respiratory Ward	77%	66/86	87%	75/86	0.078
Specialty Triage	41%	36/87	59%	51/87	0.011
2 or more post-take rounds/24 hrs	71%	60/85	87%	74/85	0.003
Formal pulmonary rehab programme	71%	62/87	84%	73/87	0.027
Access to early discharge scheme	46%	39/84	63%	53/84	0.013
Availability of acute NIV	97%	84/87	95%	83/87	0.99

*Number of hospitals varies from 84-87 according to availability of data

Table 3 shows general organisational data for the NCROP participants. 42% of respiratory departments work across more than one site. Only 75% (43/57) of those working from one site have their services located in a single area. Significant issues remain around the availability of on-site Clinical Psychology support (34%), use of specialty triage (57%), funding of smoking cessation programmes (63%), and the provision of written self-management advice to patients at the point of discharge (40%). A minority of units (13%) undertake a separate respiratory on-call rota. Units were asked to state their mean length of stay for COPD patients in their hospital and 78 did so - the median of these values was 7 days, inter-quartile range 5 to 8 days. Only 42% of units had any formal arrangements for patients with COPD to receive palliative care in their area and, of those that did not, only 51% (29/57) had any development plans for palliative care.

**Table 3:** General level of Organisation in NCROP Hospitals 2007*

**INDICATOR**	**'Met in Full'**	
Formal pulmonary rehab programme	83%	83
Access to early discharge scheme	61%	59/97
Trust Respiratory Department on a single site	58%	58
Respiratory Department in a dedicated area	64%	63/99
On-site clinical Psychology	34%	34
Written local guidelines for managing COPD	74%	74
Specialist Respiratory ward	86%	85/99
Use specialty triage	57%	57
Specialist Respiratory on-call rota	13%	13
High Dependency Unit available to COPD patients	69%	69
Funded smoking cessation programme	63%	62/99
Written self-management advice at discharge	40%	40
Local Respiratory Support Group for respiratory patients	83%	82/99
Local PCT engages with respiratory services	86%	85/99
Respiratory Interest/Network group locally	78%	76/98
Mechanism to influence local care commissioning	73%	69/95

On-site palliative care	82%	81/99
Formal arrangements for COPD patients to receive palliative care in area	42%	42/99
Policy for providing patient information about end of life care to severe COPD patients whilst in a stable state	11%	11/98
Any plans to develop/further develop palliative care services for patients with COPD	60%	59/98

*n/100 unless otherwise shown

### Non-Invasive Ventilation (NIV)

NIV provision remains excellent (Table 4). However, although there is a named lead for the service in 78% of hospitals, and the technical application and availability of NIV is good, quality issues are noted in relation to on-going staff training (56%) and the education of staff outside of specialist areas (40%). Particular areas for improvement are around the provision of information and education about NIV to patients, and detailing the ceiling of therapy. Thus, only 19% of units provide information about the indications for, and experience of, NIV, with 7% providing patients with information during the steady state. 39% of units provided written plans regarding the withdrawal of NIV. 57% of units have a weaning protocol. A third of units undertake annual audit of their NIV service. 88% of Units have written protocols for monitoring patients on NIV but only 39% of hospitals have written protocols for managing patients who fail this treatment.

**Table 4:** Non-Invasive Ventilation (NIV) quality indicators

**INDICATOR**	**'Met in Full'**	
NIV is used as the treatment of choice for persistent hypercapnoeic ventilatory failure during exacerbations despite optimal medical therapy.	80%	78/97
NIV is delivered in settings suitable for COPD patients, that is a designated area where staffs have been specifically trained in NIV. E.g. ITU, HDU, Emergency Admissions Unit or a dedicated Respiratory Ward.	74%	71/96
There is a named consultant responsible for the NIV service	78%	76/98
There is an ongoing inter-professional training programme for ALL staff involved in the care of patients established on NIV.	56%	54/96
Nurses and doctors outside of specialist respiratory wards do know how to manage patients with COPD, and are aware of the indications for and benefits of NIV.	40%	39/98
There is a written protocol that defines the monitoring of patients receiving NIV, and includes a minimum of regular clinical assessment, pulse oximetry and arterial blood gas measurements.	88%	85/97
There is a clear set of individualised written instructions for management of each patient receiving NIV, including what to do in event of deterioration and agreed ceilings of therapy	39%	38/97
Locally adapted written protocols for the management of COPD patients requiring NIV, including weaning from NIV, are available in ALL relevant clinical areas for ALL relevant staff.	57%	56/98
A selection of nasal and full face masks, types and nasal pillows are available.	55%	54/98
All areas offering NIV provide written information for patients about the indications for and patient experience of NIV.	19%	18/96
There is a policy for providing patient information about NIV to severe COPD patients whilst in a stable state e.g. in an out-patient setting or upon discharge from hospital.	7%	7/97
There is an annual audit of the use of NIV including ALL clinical areas. This audit covers both those patients offered NIV to examine its appropriate use AND those that might have benefited for NIV but who were not provided with this therapy.	32%	31/97

Percentage of NCROP sites providing NIV in 2007 that said they currently 'met in full' these indicators (denominator = number of hospitals returning data)

### Pulmonary Rehabilitation (PR)

PR programmes are comprehensive (Table 5) and widely available within the hospital setting (83%). Although PR is funded by the NHS in 88% (73/83) of units, in only 44% does funding cover sessions from a physiotherapist, dietician, social worker, pharmacist, occupational therapist, lung function technician or a previous course participant. There is a comprehensive continuation phase in only 41% of units, although 28% did state that this standard was partially met. There is a need to improve staff resuscitation training (53% trained to ALS standard) but staff to patient ratios are otherwise universally appropriate.

**Table 5:** Pulmonary rehabilitation (PR) quality indicators

**INDICATOR**	**'Met in Full'**	
The pulmonary rehabilitation programme is delivered by a fully funded, multi-disciplinary team with sessions from: a physiotherapist, dietician, social worker, pharmacist, OT, lung function technician and a previous course participant.	44%	36/82
There is a designated lead clinician and a named co-ordinator for the pulmonary rehabilitation programme.	85%	70/82
Pulmonary rehabilitation lasts a minimum of 6 weeks with exercise sessions twice a week. It is repeated regularly throughout the year.	91%	75/82
There is a continuation phase, run by people trained in pulmonary rehabilitation, in the community.	41%	34/82
The pulmonary rehabilitation programme includes education about living with COPD and ALL of the following issues: exercise, smoking cessation, diet, oxygen, social service support and benefits.	89%	72/81
Staff that supervise exercise component of the pulmonary rehabilitation programme are trained in resuscitation to Advanced Life Support standard and basic life support equipment is available (oxygen, bronchodilators and GTN) is available during these	53%	42/80
The staff/patient ratio during the exercise component of the pulmonary rehabilitation programme is at least 1:8	98%	79/81
The pulmonary rehabilitation programme provides written educational resources/leaflets for patients and carers.	95%	78/82
There are annual audits of the service that includes patients numbers AND outcomes AND patient satisfaction.	70%	57/82
Measurements such as spirometry, exercise and health status, are recorded before and after pulmonary rehabilitation.	78%	63/81

Percentage of the 83 NCROP sites providing PR in 2007 that said they 'met in full' these indicators (denominator = number of hospitals returning data)

### Early Discharge Schemes (EDS)

There has been an encouraging improvement in availability of EDS (Table 2) and good attainment of quality indicators where these schemes EDS exist (Table 6). However, 39% of units do not have an EDS and only 46% of units who do are able to enter patients from an EDS into pulmonary rehabilitation.

**Table 6:** Early Discharge service (EDS) quality indicators

**INDICATOR**	**'Met in Full'**	
There are clear written criteria for acceptance on to the early discharge scheme.	96%	55/57
The scheme is run by individuals who are capable of working proactively and independently and includes those specifically trained in respiratory medicine	93%	54/58
There is a named clinician responsible for the service	95%	55/58
There are clear written protocols of care for the management of patients under the scheme.	91%	53/58
Patients accepted for early discharge are entered onto a pulmonary rehabilitation programme: patients not accepted onto the scheme still receive a package of smoking cessation/educational support.	46%	26/57
All patients and their carers receive information about the early discharge scheme that describes what it is, and the support that is available well in advance of them needing the service.	60%	35/58
The early discharge scheme has good lines of communication to manage patient care together with their GP.	95%	55/58
The early discharge scheme runs according to the needs of the local populations.	80%	45/56
There is continuous data collection along with both prospective and annual audits of the service to monitor its effectiveness.	74%	43/58

Percentage of the 59 NCROP sites providing EDS in 2007 that said they 'met in full' these indicators (denominator = number of hospitals returning data)

### Oxygen service

Table 7 shows data for oxygen services, indicating that there are significant issues relating to availability of service, screening patients and subsequent follow-up. Thus, a long-term oxygen (LTOT) assessment service is available in 75% of units and ambulatory oxygen is provided by only 51%. LTOT assessments are undertaken by concentrator in 59% of units, there is screening for ambulatory in 48% and for short-burst in 54%. BTS criteria for follow-up are achieved for LTOT in 57% and for ambulatory oxygen in 44%. Only 58% of the hospital-based oxygen prescriptions are routed though the respiratory departments. Written information for patients is given in 66% and 43% of hospitals undertake regular audit of oxygen prescribing.

**Table 7:** Oxygen provision quality indicators

**INDICATOR**	**'Met in Full'**	
There is a Long Term Oxygen Therapy (LTOT) assessment service.	75%	74/99
There is screening in clinic of all patients with COPD to detect SaO2 <92%.	71%	70/99
The LTOT assessment includes optimising oxygen flow to achieve a PaO2 of 8 kPa or greater using arterial blood gases.	87%	84/97
The LTOT assessment uses a concentrator machine as the oxygen source.	59%	58/98
For patients prescribed LTOT, follow-up arrangements are made as recommended by the BTS guidelines for home oxygen provision	57%	56/98
There is a healthcare professional contact available to deal with queries from patients and carers concerning their oxygen therapy	82%	81/99
Ambulatory oxygen is provided by the department for suitable patients.	51%	50/98
There is screening for suitability for ambulatory oxygen, including SaO2 measurement, before referral for assessment.	48%	47/98
For patient's prescribed ambulatory oxygen, follow-up arrangements are made as recommended by the BTS guidelines for home oxygen provision.	44%	41/94
All patients receiving ambulatory oxygen receive education on how to use oxygen outside of the home.	58%	55/95
Written information is provided to all patients receiving oxygen.	66%	65/98
All hospital based oxygen prescriptions are routed through the Respiratory Department.	58%	57/98
Short Burst Oxygen is provided by the department to suitable patients.	73%	72/98
Patients are assessed for suitability before receiving Short Burst Oxygen.	54%	52/96
Regular audits of oxygen prescribing are carried out.	43%	42/98

Percentage of the 100 NCROP sites providing oxygen in 2007 that said they 'met in full' these indicators (denominator = number of hospitals returning data)

## Discussion

Hospitals recruited to the NCROP were typically larger Units that had participated in previous national audit. This baseline survey showed that useful improvements in COPD services had occurred since 2003. In particular, there was an increase in the number of early discharge schemes and excellent provision of NIV, the latter comparing favourably to other surveys [2,3]. However, wide variations in management again remained, notably around the organisation of acute care, oxygen services, provision of written information to patients, patient self-care and the continuation phase of pulmonary rehabilitation. There were issues relating to staff training, provision of palliative care and hospitals tended not to audit key aspects of their own service.

We did not seek to identify drivers for change since 2003, merely to document and to assess the organisation of services amongst NCROP hospitals at the inception of this project. An education programme had been introduced after the 2003 UK COPD Audit but its' impact not formally assessed. One could speculate that repeated surveying may itself lead to improvements but there has also been continuing investment and reorganisation within the Primary and Secondary Care sectors of UK health care over the last 5 years. Extra resources, improved clinical/managerial leadership or a combination of these factors may be relevant.

The notable increase in early discharge schemes may have arisen in part for economic reasons because they are attractive to fund in an NHS environment where there is great pressure on acute beds and an increasing emphasis on community care. The BTS has also published useful guideline documents for both NIV and Early Discharge schemes [4,5] that have been widely supported by colleagues across the UK.

There remain significant gaps in service despite repeated cycles of audit and a useful evidence base for clinicians and managers to draw upon in areas such as acute care [6,7], hospital at home [5], patient self-care [8], the provision of information to patients [9-11] and information about the costs of managing COPD [12,13]. The NCROP partner organisations were already aware of a need to test alternative methods for generating change, and this formed the rationale for the NCROP which investigates, in a controlled fashion, the effect of multi-disciplinary Peer Review on service development and organisation within recruited hospitals.

We planned to pair hospitals in rough geographical proximity according to the outcome of their baseline survey results, those with good scores ideally meeting those who are lacking. Each hospital within a pair would undertake a structured Peer Review of the other's COPD service, the visiting team comprising both clinicians and managers. They would prepare a standardised report, containing key areas for change, the final draft having Executive "sign-off" and approval of the development plan. We postulated that this intervention, by virtue of its' structure and manager-clinician involvement, would accelerate the change process. The baseline survey would be repeated initially one year after the intervention, with comparison between intervention and control sites. The final data outlining changes within the intervention and control groups over time will be reported separately.

There are clearly limitations of surveys and studies such as this; inevitably, participating hospitals are likely to be those with "enthusiasts", as evidenced by the high concordance between participants in both 2003 and 2007 surveys. Smaller, less well organised or resourced units are more likely to be missed, despite the known sense of worth obtained from participation in National audit [14] and the likelihood that there services will be less well-organised [15]. However, our 100 recruited hospitals represent a good cross-section of both Teaching and District General Units across the UK.

There are also many confounding factors that may potentially influence the organisation of care during the lifetime of the study. While change is often slow, and a repeat survey to assess change 12 months after the intervention could be considered rather soon, we are aware that both National and local priorities can change rapidly in the UK National Health Service. There is also a fast-developing and powerful system of Practice-Based Commissioning in England [16] that is shifting COPD care and resources from hospitals into Primary Care. The system has not developed uniformly across the country but will doubtless influence change in hospital services where it is being introduced. We hope, therefore, to continue surveying our recruited Units beyond 1 year after the original Peer Review Intervention in order to account for some of these factors.

## Conclusion

In summary, we have found welcome improvements in COPD care since 2003, but significant organisational and clinical issues remain. That such shortfalls in care still occur despite repeated audit emphasises how important it is to understand not only the reasons but also to find better ways of hastening change. This poses a great challenge for investigators working in a healthcare landscape that is itself subject to constant change. We hope the NCROP will improve our understanding of some of the specific factors which facilitate or hinder change within hospitals providing acute COPD care.

## Competing interests

The authors declare that they have no competing interests.

## Authors' contributions

RAS was associate director of the project, participated in the design and drafted the manuscript. BDWH provided medical leadership, participated in project design and assisted with the manuscript. DL undertook the statistical analysis and contributed to the manuscript. RJB was lead project manager and helped with project design. NAP was assistant project manager. HSRH provided medical leadership and helped with project design. JMP provided medical leadership and helped with project design. CMR conceived and directed the project, contributing also to the manuscript. All authors read and approved the final manuscript.

## Pre-publication history

The pre-publication history for this paper can be accessed here:


